# The Significance of Serum CA-125 Level on the Live Birth Rates of In Vitro Fertilisation in Women with Endometriosis

**DOI:** 10.3390/medicina62010053

**Published:** 2025-12-26

**Authors:** Hoi Ki Chung, Shui Fan Lai, Rebecca Siu Fan Wan, Jennifer Ka Yee Ko, Ernest Hung Yu Ng, Raymond Hang Wun Li

**Affiliations:** 1 Department of Obstetrics and Gynaecology, Princess Margaret Hospital, Hong Kong; chk512@ha.org.hk (H.K.C.); wr277@ha.org.hk (R.S.F.W.); 2 Department of Obstetrics and Gynaecology, Kwong Wah Hospital, Hong Kong; lsf087@ha.org.hk; 3 Department of Obstetrics and Gynaecology, School of Clinical Medicine, LKS Faculty of Medicine, The University of Hong Kong, Queen Mary Hospital, Hong Kong; kky381@ha.org.hk (J.K.Y.K.); nghye@hku.hk (E.H.Y.N.)

**Keywords:** CA-125 antigen, endometriosis, in vitro fertilization, live birth rate

## Abstract

*Background and Objectives*: To evaluate the significance of serum CA-125 level on the live birth rate of in vitro fertilization (IVF) in women with endometriosis. *Materials and Methods*: This retrospective observational study included women with endometriosis who underwent one stimulated IVF cycle. Serum CA-125 levels were measured in archived serum samples collected prior to ovarian stimulation, on the day of ovulation trigger, and during frozen embryo transfer. Serum CA-125 levels were compared between cycles with and without a live birth in both stimulated IVF and frozen embryo transfer cycles, with a subgroup analysis using a cut-off of 35 IU/L. *Results*: Within the same patient undergoing the same IVF cycle, serum CA-125 level on the trigger day of the stimulated cycle was significantly lower than the baseline level before stimulation started (35.1 IU/L [21.0–64.5 IU/L] vs. 46.8 IU/L [25.9–104.0 IU/L], *p* < 0.001), but was higher than that in the frozen embryo transfer cycles (31.7 IU/L [19.9–58.7 IU/L] vs. 27.3 IU/L [18.1–59.9 IU/L], *p* = 0.041). Serum CA-125 levels were not associated with the live birth rate in the stimulated IVF cycle and frozen embryo transfer cycles. In subgroup analysis, women with serum CA-125 level ≥ 35 IU/L on the trigger day had a significantly higher pregnancy rate from the fresh embryo transfer cycle than those with level < 35 IU/L (adjusted odds ratio 4.126, 95% CI 1.241–13.720, *p* = 0.021). The cut-off of 35 IU/L did not show significant differences in live birth rate for either stimulated or frozen embryo transfer cycles. *Conclusions*: In women with endometriosis, no significant differences in serum CA-125 levels were found between those with and without a live birth in fresh and frozen embryo transfer cycles. In fresh embryo transfer cycles, those with serum CA-125 ≥ 35 IU/L had significantly higher pregnancy rates.

## 1. Introduction

Endometriosis is a benign gynaecological disease affecting 10% of reproductive age women and up to 50% of women who experience infertility. It is characterised by the presence of endometrial stromal and glandular tissue outside the uterine cavity. It can seriously affect one’s quality of life and cause infertility. Endometriosis can lead to pelvic anatomical distortion via chronic inflammation and may negatively impact the quality of oocytes, as well as endometrial receptivity [1].

Endometriosis remains a challenge to women undergoing in vitro fertilisation (IVF) as it is associated with a lower oocyte yield per cycle [2,3,4,5]. A meta-analysis reported comparable live birth rates among women with intact endometrioma, peritoneal endometriosis and surgically treated endometrioma [4]. A recent prospective observational study consisting of 1040 women, however, showed a lower cumulative live birth rate in women with deeply infiltrating endometriosis and/or endometrioma despite no difference in the number of retrieved mature oocytes, fertilisation rate, or number of good quality embryos [6]. Some studies suggested that disease stages of endometriosis had an impact on the pregnancy outcome of IVF [5,7,8].

Besides imaging and laparoscopy, there is an increasing interest in serum biomarkers for predicting the severity and prognosis of endometriosis. The diagnostic performance of more than 100 biomarkers has been investigated for endometriosis. CA-125 is a glycoprotein expressed by coelomic epithelium including that of the endometrium, fallopian tube, ovary, and peritoneum, and has been extensively studied in endometriosis. Its serum level is higher in cases of more advanced disease staging and decreases after surgical treatment [9,10,11,12,13,14,15,16].

The significance of serum CA-125 level on IVF outcomes has been evaluated, but the results were inconsistent [17,18,19] and often focused on non-endometriosis populations [20,21,22]. Some studies suggested that serum CA-125 level was not useful in predicting ovarian response and IVF outcomes [17,20,21,22], but their sample sizes were small, ranging from 33 to 74 subjects. Chryssikopoulos et al. showed that serum CA-125 level significantly increased from the ovulation trigger day to the oocyte retrieval day and decreased on the embryo transfer day in those who became pregnant from IVF cycles using a gonadotrophin releasing hormone (GnRH) agonist protocol [18]. Tavmergen et al. also demonstrated pregnant women exhibited higher CA-125 levels on the day before trigger, on trigger day, and on the oocyte retrieval day [23]. However, the above studies adopted the GnRH agonist protocol for ovarian stimulation and investigated the fresh embryo transfer cycles only, including a limited number of endometriosis cases. The GnRH agonist protocol is now much less frequently used in our locality compared to the antagonist protocol, primarily due to its shorter medication duration and lower risk of ovarian hyperstimulation syndrome, with a comparable live birth rate [24]. Additionally, the agonist protocol may also lead to a reduction in serum CA-125 levels.

The effect of ovarian stimulation on serum CA-125 levels and the significance of serum CA-125 level on the live birth rate of IVF in women with endometriosis remains unclear. Therefore, this retrospective study aimed to address the following questions in women with endometriosis: (1) whether there was any difference in serum CA-125 level before and after ovarian stimulation; (2) whether there was any difference in serum CA-125 level between a stimulated IVF cycle and a subsequent frozen embryo transfer cycle within the same patients; and (3) whether there was any effect of serum CA-125 levels on the live birth rate of the stimulated IVF cycle and the frozen embryo transfer cycle in women with endometriosis.

### Plain Language Summary

Endometriosis is a benign condition that affects up to 50% of women with infertility. There is evidence suggesting that endometriosis may adversely affect in vitro fertilization (IVF) success in various ways, including reducing the number and quality of eggs retrieved and affecting the receptiveness of the uterine lining.

CA-125 is a well-known marker found in the blood that is associated with gynaecological conditions such as ovarian malignancy and endometriosis. The connection between CA-125 levels and IVF outcomes, specifically regarding pregnancy and live birth rates, remains uncertain. This retrospective study aimed to explore the relationship between CA-125 levels and live birth rates in women undergoing IVF treatment for endometriosis. The findings indicated that CA-125 levels did not correlate with overall pregnancy or live birth rates. However, in fresh embryo transfer cycles, women with CA-125 levels of 35 IU/L or higher had significantly higher pregnancy rates.

## 2. Materials and Methods

This was a retrospective observational study. Women who were known to have endometriosis and underwent one stimulated cycle in the assisted reproduction units in Queen Mary Hospital and Kwong Wah Hospital, Hong Kong, from 2021 to 2023, were recruited into the study. The study was approved by the Central Institutional Review Board of Hospital Authority and the Institutional Review Board of the University of Hong Kong/Hospital Authority Hong Kong West Cluster. (Ref no. CIRB-2024-042-4/UW 24-384) This study was conducted in conformance to the Declaration of Helsinki.

Endometriosis was diagnosed either histologically following abdominal or laparoscopic surgery or sonographically where an endometriotic cyst was shown on pelvic scanning. Those aged over 43 years old, suffering from premature ovarian insufficiency or malignancy, and whose archived serum samples could not be retrieved were excluded from the analysis.

Women who had one stimulation cycle and subsequently underwent subsequent frozen embryo transfer were recruited in the analysis. Serum CA-125 levels were measured from the archived serum samples previously taken in the early follicular phase at commencement of ovarian stimulation and on the trigger day in the stimulated IVF cycles, as well as on the luteinising hormone (LH) surge day or the day of starting progestogen in their first frozen embryo transfer in natural cycles or hormone replacement cycles, respectively, where applicable. Informed written consent was obtained from all subjects prior to the start of IVF to donate the surplus serum sample for research use. These serum samples were frozen at −20 °C in the laboratory. The archived serum samples were retrieved and assayed for serum CA-125 by a chemiluminescence method (ARCHITECT CA125, Abbott Laboratories, Abbott Park, IL, USA; catalogue number 02K4529). The sensitivity of the assay is ≤1.0 U/mL, and the total and within-run coefficients of variation are ≤4.3% and ≤3.2% respectively.

In this study, women received either human menopausal gonadotrophin or recombinant FSH for ovarian stimulation in either GnRH antagonist protocol or progestin-primed ovarian stimulation (PPOS) protocol. The initial dose of gonadotrophin (Gonal-F^®^, Merck, Rome, Italy or Menopur^®^, Ferring, Kiel, Germany), ranging from 150 IU to 300 IU per day, was determined according to the antral follicle count and body weight of the women. In the GnRH antagonist protocol, ganirelix (Orgalutran^®^, NV Organon, Oss, The Netherlands) or cetrorelix (Cetrotide^®^, Baxter, Saale, Germany) was given starting from the sixth day of stimulation till the day of trigger. In PPOS protocol, medroxyprogesterone acetate (Provera^®^, Pfizer, Milano, Italy) 10 mg daily was started from the first day of commencing gonadotrophin stimulation till the day of trigger.

Human chorionic gonadotrophin (HCG) (Ovidrel^®^ 250 μg, Merck, Rome, Italy) was administered to trigger oocyte maturation when at least three leading follicles reached a mean diameter of 17 mm or above. Transvaginal ultrasound-guided oocyte retrieval was performed 36 h after the HCG administration. Fertilisation was achieved through conventional insemination or intracytoplasmic sperm injection, depending on semen quality. Decision on extended embryo culture depended on the number of embryos available on day 2 or based on patients’ preference after counselling. One cleavage stage embryo or blastocyst was replaced using a soft catheter (Sydney IVF Embryo Transfer Catheter^®^, Cook, Bloomington, IN, USA) under transabdominal ultrasound guidance. Fresh embryo transfer was not performed if the woman received the PPOS regimen, or was considered at risk of ovarian hyperstimulation syndrome, or had serum estradiol concentration on the day of HCG trigger exceeding 20,000 pmol/L. Day 2 cleavage-stage embryos or blastocysts were cryopreserved using a vitrification technique.

Frozen embryos or blastocysts were transferred at least one month after the stimulated IVF cycle in natural cycles in ovulatory women, or in letrozole-induced or hormone replacement cycles in anovulatory women. For natural cycle frozen embryo transfer, follicular tracking was performed 18 days before the next expected date of menstruation. Serum LH was checked daily when the leading follicle reached a mean diameter > 14 mm. The LH surge was defined as serum LH level above 20 IU/L and more than doubled the average value of the previous 3 days. For hormone replacement cycles, endometrial thickness was measured after 2 weeks of oral estradiol (Estrofem^®^, Novo Nordisk, Copenhagen, Denmark) administration. When the endometrial thickness reached > 7 mm, oral dydrogesterone (Duphaston^®^, Abbott, Weesp, The Netherlands) and vaginal progesterone (Endometrin^®^, Ben-shimon, Tel Aviv, Israel) were added, and transfer was scheduled according to the stage of embryo or blastocyst.

A urine pregnancy test was performed 18 days after the HCG trigger in fresh embryo transfer cycles, or after the LH surge day or starting progestogen in frozen embryo transfer cycles. Pelvic ultrasound scan was arranged 2 weeks later, i.e., at 6 weeks of gestation in those with a positive urine pregnancy test to check the location and number of intrauterine gestational sac(s) and foetal viability. The women were referred out for antenatal care when the repeated scan at 8 weeks’ gestation confirmed an ongoing pregnancy.

Demographic information, including age of women, body mass index, duration of infertility, anti-Mullerian hormone level, previous endometriosis surgery, presence and size of endometrioma, and details of IVF cycles including type of ovarian stimulation regimen, total dose of gonadotropin given, peak serum estradiol level, number of oocytes aspirated/fertilized, number of utilizable embryos/blastocysts, number of embryo or blastocyst replaced and pregnancy outcomes were extracted from the assisted reproduction database.

The key outcomes of the study were the live birth rate and serum CA-125 levels at different phases of the IVF cycle. A live birth was defined as the birth of a viable infant after 24 weeks of gestation. Clinical pregnancy was defined by the presence of an intrauterine gestational sac. IVF outcome was tracked using the Hospital Authority electronic patient record system or through reply slips received from the women or their obstetricians. Women were contacted by research nurses after their expected date of delivery if no reply letter was received or if the delivery did not occur in a public hospital.

During preparation of this manuscript, AI-assisted technology, namely ‘POE’, was used in the writing progress to improve readability. After using this tool, the authors reviewed and edited the content as needed and take full responsibility for the content of the publication.

### Analysis

Serum CA-125 levels in the early follicular phase (baseline level) were compared against the level on the trigger day in the stimulated IVF cycle and the level on the LH surge day or the day of starting progestogen in the frozen embryo transfer cycles (level in the mid-cycle). The association of serum CA-125 level and live birth was studied. A subgroup analysis of serum CA-125 level < 35 IU/L vs. ≥35 IU/L was also carried out.

Analyses were performed using SPSS statistical software (Windows version 27; IBM Corp., Armonk, NY, USA). Spearman’s correlation and Mann–Whitney U test were used for continuous data and the Chi-square test or Fisher’s exact test were used for categorical data. The predictive role of serum CA-125 level on IVF outcomes was analyzed in a binary logistic regression model after controlling for antral follicle count. A *p* value of <0.05 was considered statistically significant.

## 3. Results

### 3.1. Demographic Characteristics

**Figure 1:**
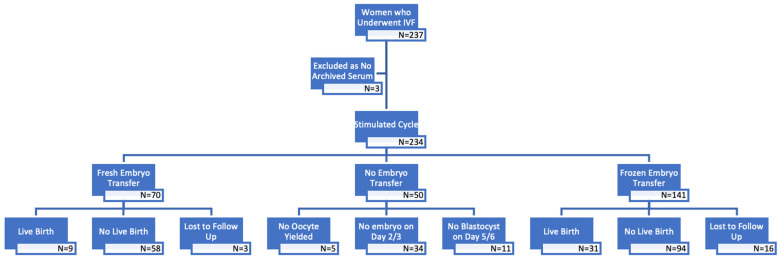
Flow chart of patients in this study.

A total of 234 women were recruited in the study, with 207 cycles reporting their pregnancy outcomes (Figure 1). In the diagnosis of endometriosis, 69.7% of women were diagnosed through surgery, while 30.3% were diagnosed using ultrasonography. Among those with a baseline serum CA-125 level of ≥35 IU/L, there was a higher proportion of women with endometriomas during ovarian stimulation, and these were generally larger in size. However, the proportion of women with a history of endometriosis surgery was comparable in both subgroups (Table 1).

### 3.2. Serum CA-125 Levels During Stimulated IVF and Frozen Embryo Transfer Cycles

During the stimulated cycle, serum CA-125 level on the trigger day of the stimulated cycle was significantly lower than the baseline level (35.1 IU/L [21.0–64.5 IU/L] vs. 46.8 IU/L [25.9–104.0 IU/L, respectively, *p* < 0.001). The serum CA-125 level on the trigger day was significantly lower in women using the PPOS protocol compared to those using the GnRH antagonist protocol (29.1 IU/L [16.3–55.1 IU/L] vs. 39.8 IU/L [21.8–74.6 IU/L, respectively, *p* = 0.004) (Supplementary Table S1).

For women who had frozen embryo transfer, serum CA-125 level on the trigger day in the stimulated cycle was significantly higher than that at mid-cycle (i.e., on the day of LH surge or starting progestogen) in the frozen embryo transfer cycle, within the same patients (31.7 IU/L [19.9–58.7 IU/L] vs. 27.3 IU/L [18.1–59.9 IU/L], respectively, *p* = 0.041). Among natural cycle and hormone replacement cycles, the CA-125 level at the LH surge day or the day of starting progestogen was not significantly different (25.11 IU/L [16.89–61.68 IU/L] vs. 29.87 [19.91–66.00 IU/L], *p* = 0.325).

### 3.3. Association of Serum CA-125 Levels and Live Birth in Fresh and Frozen Embryo Transfer Cycles

There were nine live births in those having fresh embryo transfer cycles and 31 in those undergoing frozen embryo transfer cycles, respectively. Age of women, body mass index, duration of infertility, antral follicle count, types of cycle, anti-Mullerian hormone level, size of pre-existing endometrioma, serum CA-125 level at baseline and on the trigger day or at mid-cycle were similar between cycles with or without a live birth in fresh embryo transfer (Table 2) and frozen embryo transfer cycles (Table 3).

The demographic characteristics, including age, body mass index and duration of infertility and the ovarian response did not differ significantly between the groups with serum CA-125 level < 35 IU/L and ≥35 IU/L on trigger day in the stimulated IVF cycle (Table 4) and at mid-cycle of the frozen embryo cycle (Table 5). In univariate analyses, antral follicle count was positively associated with the occurrence of pregnancy (*p* = 0.047). In the multivariate binary logistic regression analysis, serum CA-125 level *≥* 35 IU/L on the trigger day was associated with a significantly higher pregnancy rate (adjusted odds ratio 4.126, 95% confidence interval 1.241–13.720, *p* = 0.021) in the fresh transfer cycles only, after controlling for antral follicle count (Supplementary Table S2).

### 3.4. Effect of Endometriosis Surgery on Serum CA-125 and Anti-Mullerian Hormone (AMH) Levels

Among women with a history of endometriosis surgery, the antral follicle counts, serum CA-125 levels at baseline and on trigger day, oocyte retrieval, stimulation protocols and fresh embryo transfer rates were comparable to those of women with no prior surgery (Table 6). However, AMH levels were significantly lower in women who underwent endometriosis surgery (1.31 ng/mL [0.73–2.35 ng/mL] vs. 2.3 ng/mL [1.08–3.99 ng/mL], *p* = 0.013). Total dose of gonadotrophins used was significantly higher in the surgery group (3000 IU [2475–3600 IU] vs. 2700 IU [2250–3300 IU], *p* = 0.040). Previous endometriosis surgery was positively associated with the live birth rate in the stimulated cycle in the univariate analysis (*p* = 0.028) but not in the logistic regression (*p* = 0.998), after controlling for antral follicle count.

Additionally, AMH levels were not associated with serum CA-125 levels at baseline (r = −0.22, *p* = 0.806) and on trigger days (r = −0.29, *p* = 0.744), as well as with the size of existing endometriomas. (r = 0.093, *p* = 0.308).

## 4. Discussion

### 4.1. Key Findings

Serum CA-125 level on the trigger day of the stimulated cycle was significantly lower than the baseline level but was significantly higher than that at mid-cycle in the frozen embryo transfer cycle within the same patients. Serum CA-125 levels were not associated with the live birth rate of the stimulated IVF cycle and frozen embryo transfer cycles. The difference in serum CA-125 levels between stimulated cycle and frozen embryo transfer cycle was small and may not have clinical significance.

Serum CA-125 levels at baseline or on the trigger day were not associated with the live birth rate in the stimulated IVF cycle, while serum CA-125 levels at mid-cycle were not associated with the live birth rate in frozen embryo transfer cycles. In subgroup analysis, a serum CA-125 level ≥ 35 IU/L on the trigger day was associated with significantly higher pregnancy rates in fresh embryo transfer cycles but no such difference was demonstrated in frozen embryo transfer cycles.

AMH levels were significantly lower in women who had previous endometriosis surgery. After excluding existing endometriomas during the stimulation cycle, the serum CA-125 levels at baseline and on trigger day were similar for women with and without prior surgery. Additionally, there was no association between endometriosis surgery and live birth rates in the stimulated cycles in the logistic regression analysis.

### 4.2. Change in Serum CA-125 Level During the Stimulated IVF Cycle

Previous research indicated that serum CA-125 levels in healthy premenopausal women were significantly higher during the follicular phase, followed by a decrease in the remainder of the cycle [25,26,27]. It is thought that the CA-125 enters the bloodstream through the breakdown of the cervical mucus barrier or retrograde menstruation, leading to a rise in serum level, and dampens when menstrual bleeding ceases.

There is controversy about serum CA-125 level variations in stimulated cycles. Studies investigating the trend of serum CA-125 level during stimulated cycles were limited and had inconsistent results. While some studies observed a rise in serum CA-125 level following the trigger day [17,18,22], others revealed that serum CA-125 levels remained stable during the stimulation cycle [28,29]. Hauzman et al. observed a decrease in serum CA-125 level from the start of stimulation to oocyte retrieval only in pregnant cycles [20]. However, it is worth noting that aforementioned studies reporting the trends of serum CA-125 level during ovarian stimulation used the GnRH agonist protocol, whereas our study primarily involved the use of the GnRH antagonist or PPOS regimens. Moreover, it is not certain whether pituitary suppression by GnRH agonist would have influenced the systemic CA-125 secretion. During ovarian stimulation in the current study, CA-125 level decreased compared to baseline, suggesting that developing follicles were not the primary source of CA-125. In the GnRH antagonist or PPOS protocols, the antagonist or progestin serves to suppress LH surge and, consequently, ovulation, mimicking the follicular phase of a spontaneous menstrual cycle. This may explain why the CA-125 level was significantly dropped in the GnRH antagonist or PPOS regimen. Additionally, the serum CA-125 level on the trigger day was significantly lower in the PPOS group. This effect may be attributed to the stabilizing impact of progestin on the endometriotic tissue.

In this study, serum CA-125 levels at both baseline and on trigger day were high (≥35 IU/L), with median levels of 46.8 IU/L (25.9–104.0 IU/L) and 35.1 IU/L (21.0–64.5 IU/L), respectively. This finding aligned with prior studies that reported elevated serum levels during menstruation in women with endometriosis [30,31]. The exaggerated rise in CA-125 levels during spontaneous menstruation is thought to result not only from menstruation itself but also contributed by endometriosis. It has been suggested that the disintegration of peritoneal implants during menstruation results in a local inflammatory reaction and peritoneal irritation, which may in turn cause increased CA125 shedding into the circulation [30]. 

### 4.3. Effect of Serum CA-125 Level on the Ovarian Response and Pregnancy Rate

Despite comparable age and antral follicle counts in both high and low serum CA-125 subgroups, our study found no association of serum CA-125 levels with ovarian response parameters, such as total dose of gonadotropin given, peak estradiol level and number of oocytes retrieved, in line with previous studies [17,19,22]. Wang et al. demonstrated that the ovarian responses were similar between endometriosis and control groups [32]. This implies that the serum CA-125 level may have no predictive role in ovarian response in women with endometriosis.

We observed that serum CA-125 level on the trigger day ≥ 35 IU/L was associated with a significantly higher pregnancy rate in the IVF cycle with fresh embryo transfer. Previous studies on the prognostic value of serum CA-125 level, which primarily focused on women without endometriosis, reported mixed results. Some concluded that serum CA-125 level did not have a predictive value for IVF outcome in agonist cycles [17,22] and in long protocol, flare protocol and GnRH antagonist protocol [33]. In other studies, however, serum CA-125 level in the stimulated cycle was significantly higher in women who attained pregnancy than those who did not, in both endometriosis and non-endometriosis cases, suggesting a role for serum CA-125 level in predicting pregnancy outcome [19]. Baalbergen et al., found that in non-endometriosis cases, serum CA-125 level rose post-oocyte retrieval, with a greater rise after successful implantation, and higher serum CA-125 level in the pregnant patients 14 days after embryo transfer [34]. The authors proposed that serum CA-125 level might be an indicator of a better endometrial receptivity in pregnant women. Elevated serum CA-125 levels might indicate the status of endometriosis and, indirectly, the level of inflammation. Increased inflammation factors may enhance the perfusion of gynecological organs, thereby improving the receptivity of the endometrium, but thus postulation needs proof by mechanistic studies. In view of these contradicting results, further large-scale prospective studies focusing on endometriosis cases are needed to ascertain the association between serum CA-125 at different stages and successful pregnancy establishment, potentially informing treatment strategies.

### 4.4. Effect of Endometriosis Surgery on Serum CA-125 Levels and Outcomes

In the current study, women with a history of endometriosis surgery exhibited significantly lower AMH levels and required higher doses of gonadotropins, with no differences in oocyte yield or live birth rates in stimulated cycles. These findings align with a previous review [35]. Ovarian cystectomies inevitably remove some healthy follicles, and electrosurgery damages ovarian tissue, leading to reduced AMH levels in the operated group and poorer response to gonadotropins. Considering the latest evidence, along with women’s age, symptoms, the number of prior ovarian surgeries, ovarian reserve, and the size and bilaterality of endometriomas, is essential for counselling regarding endometriosis surgery prior to fertility treatment.

### 4.5. Limitations

First, this is a retrospective study with a small sample size. While it is comparatively larger than many of the previously reported studies, only 9 live births occurred in the fresh embryo transfer cycle. This may limit the statistical power to determine the association, even if it is present. Second, not all of our patients were diagnosed with endometriosis surgically and/or histologically, which should be the gold standard of diagnosing endometriosis. In clinical practice, not all women undergoing IVF had prior surgery. This may result in a bias towards the more severe symptomatic cases of endometriosis in our cohort. There may also introduce misclassification bias and dilute the observed effect of serum CA-125, since the presence of endometrioma does not indicate active peritoneal endometriosis. Third, the staging of endometriosis was not evaluated, which enables confirmation of its association with CA-125 levels, thereby strengthening the validity of the study hypothesis. Despite that, the presence and size of endometriomas during ovarian stimulation can also reflect disease status, which current study showed to have a strong association with serum CA-125 levels.

## 5. Conclusions

In women with endometriosis, serum CA-125 levels on the trigger day were significantly lower than the baseline levels during a stimulated IVF cycle but significantly higher than that in frozen embryo transfer cycles. No significant differences in serum CA-125 levels were found between those with and without a live birth in both fresh and frozen embryo transfer cycles. In fresh embryo transfer cycles, those with serum CA-125 ≥ 35 IU/L had significantly higher pregnancy rates.

## Figures and Tables

**Table 1:** Characteristics and pregnancy outcome between groups with different baseline serum CA-125 level.

Parameters	All (n = 234)	<35 IU/L (n = 90)	≥35 IU/L (n = 144)	*p*-Value
Age of women (years)	36.0 (34.0–37.0)	35.0 (33.5–37.0)	36.0 (34.0–37.0)	0.610
Body mass index (kg/m^2^)	21.2 (19.6–23.0)	20.7 (19.5–22.5)	21.5 (19.7–23.3)	0.136
Duration of infertility (years)	3.0 (2.0–5.0)	3.0 (2.0–5.0)	3.0 (2.0–5.0)	0.818
Antral follicle count	7.0 (5.0–11.0)	8.0 (5.0–12.0)	7.0 (4.0–11.0)	0.797
Anti-Mullerian hormone (ng/mL)	1.5 (0.8–2.7)	1.5 (0.8–2.7)	1.6 (0.8–2.8)	0.812
Diagnosis of endometriosis				
By operation	163/234 (69.7%)	60/90 (66.7)	103/144 (71.5)	0.555
By sonography	71/234 (30.3%)	30/90 (33.3)	41/144 (28.5	
Previous endometriosis surgery	152/234 (65.0)	52/90 (57.8)	100/144 (69.4)	0.155
Presence of endometrioma during ovarian stimulation	129/216 (59.1%)	36/77 (46.8%)	93/139 (66.9%)	0.009 *
Size of endometrioma if any (mm)	16.0 (0–26.0)	0 (0–22.5)	18.5 (0–27.0)	0.005 *
Ovarian stimulation protocol				0.315
GnRH antagonist	157 (68.0%)	57 (64.0%)	100 (70.4%)	
PPOS	74 (32.0%)	32 (36.0%)	42 (29.6%)	
Total dose of gonadotropins (IU)	3000 (2400–3600)	2925 (2250–3525)	2963 (2456–3600)	0.682
Peak serum estradiol level (pmol/L)	12,334 (7007–17,763)	12,852 (7270–17,737)	17,793 (6833–17,836)	0.532
Number of oocytes retrieved	6.0 (4.0–11.0)	7.0 (4.0–12.5)	6.0 (3.0–10.0)	0.081
Pregnancy rate in the fresh IVF cycle	20/70 (28.6%)	6/26 (23.1%)	14/44 (31.8%)	0.585
Live birth rate in the fresh IVF cycle	9/67 (13.4%)	3/25 (12.0%)	6/42 (14.3%)	0.762

Values are expressed as median (25–75th percentile) for continuous variables and as number (percentage) for categorical variables. PPOS: progestin-primed ovarian stimulation. * Statistically significant.

**Table 2:** Demographic characteristics, serum CA-125 level and stimulation parameters in fresh embryo transfer cycles.

Parameters	Attainment of Live Birth		*p*-Value
	Yes (n = 9)	No (n = 58)	
Age of women (year)	37.0 (33.0–38.0)	36.0 (34.0–38.0)	0.492
Body mass index (kg/m^2^)	22.7 (20.3–23.6)	21.5 (20.0–24.5)	0.455
Duration of infertility (years)	4.0 (2.5–5.0)	4.0 (2.0–5.0)	0.800
Antral follicle count	7.0 (4.5–8.0)	6.0 (4.0–8.0)	0.625
Anti-Mullerian hormone (ng/mL)	1.7 #	1.2 (0.7–2.3)	0.667
Presence of endometrioma during ovarian stimulation	5/9 (55.6%)	35/54 (64.8%)	0.593
Size of endometrioma if any (mm)	18.0 (0–22.0)	19.5 (0–30.0)	0.290
Baseline serum CA-125 level (IU/L)	55.4 (23.0–81.1)	45.8 (28.7–104.5)	0.971
Baseline serum CA-125 level ≥ 35 IU/L	6 (66.7%)	35 (60.3%)	1.000
Serum CA-125 level on the trigger day (IU/L)	43.5 (18.5–75.5)	32.0 (21.6–70.4)	0.815
Serum CA-125 level on the trigger day ≥ 35 IU/L	6 (66.7%)	25 (43.1%)	0.287
Total dose of gonadotropins (IU)	3300 (2138–3450)	3038 (2644–3600)	0.530
Peak serum estradiol level (pmol/L)	9596 (6444–14,547)	8682 (4883–13,903)	0.429
Number of oocytes retrieved	8.0 (6.0–11.5)	8.0 (5.0–12.0)	0.993

Values are expressed as median (25–75th percentile) for continuous variables and as number (percentage) for categorical variables; # 25–75th percentile not available as only two values for analysis.

**Table 3:** Demographic characteristics, serum CA-125 level and stimulation parameters in frozen embryo transfer cycles.

Parameters	Attainment of Live Birth		*p*-Value
	Yes (n = 31)	No (n = 94)	
Age of women at the time of transfer (year)	36.0 (33.0–37.0)	36.0 (34.0–37.0)	0.605
Body mass index (kg/m^2^)	20.9 (19.6–23.4)	21.1 (19.5–22.7)	0.878
Duration of infertility (years)	4.0 (2.0–5.0)	3.0 (2.0–5.0)	0.830
Antral follicle count	10.0 (6.5–13.0)	9.0 (6.0–13.0)	0.376
Anti-Mullerian hormone (ng/mL)	1.9 (0.9–2.5)	1.9 (1.0–2.9)	0.789
Endometrial thickness	9.9 (8.6–12.4)	10.7 (9.5–12.5)	0.230
Type of cycles			0.833
Natural cycle	19/31 (61.3)	55/94 (58.5)	
Hormone replacement	10/31 (32.3)	35/94 (37.2)	
Letrozole-induced	2/31 (6.5)	4/94 (4.3)	
Baseline serum CA-125 level (IU/L)	31.4 (23.3–84.3)	42.6 (23.2–105.1)	0.515
Baseline serum CA-125 level ≥ 35 IU/L (%)	15 (48.4%)	53 (56.4%)	0.534
Serum CA-125 level on day of LH surge or starting progestogen (IU/L)	23.9 (20.3–46.9)	32.2 (17.3–70.7)	0.498
Serum CA-125 level on day of LH surge or starting progestogen ≥ 35 IU/L (%)	10 (32.3%)	42 (45.7%)	0.293

Values are expressed as median (25–75th percentile) for continuous variables and as number (percentage) for categorical variables.

**Table 4:** Characteristics and pregnancy outcome between groups with serum CA-125 level on the trigger day of IVF.

Parameters	All (n = 234)	<35 IU/L (n = 117)	≥35 IU/L (n = 117)	*p*-Value
Age of women (years)	36.0 (34.0–37.0)	36.0 (33.0–37.0)	36.0 (34.0–37.0)	0.453
Body mass index (kg/m^2^)	21.2 (19.6–23.0)	20.7 (19.5–22.6)	21.7 (19.9–23.2)	0.083
Duration of infertility (years)	3.0 (2.0–5.0)	3.0 (2.0–5.0)	3.0 (2.0–5.0)	0.818
Antral follicle count	7.0 (5.0–11.0)	7.5 (5.0–11.0)	7.0 (4.0–11.25)	0.880
Anti-Mullerian hormone (ng/mL)	1.5 (0.8–2.7)	1.3 (0.8–2.6)	1.7 (0.8–2.9)	0.372
Presence of endometrioma during ovarian stimulation	129/216 (59.7%)	49/102 (48.0%)	80/114 70.2%)	0.001 *
Size of endometrioma if any (mm)	16.0 (0–26.0)	0 (0–22.3)	19.0 (0–27.0)	0.001 *
Ovarian stimulation protocol				0.003 *
GnRH antagonist	160 (68.4%)	69 (59.0%)	91 (77.8%)	
PPOS	74 (31.6%)	48 (41.0%)	26 (22.2%)	
Total dose of gonadotropins (IU)	3000 (2400–3600)	3000 (2325–3600)	2963 (2475–3356)	0.998
Peak serum estradiol level (pmol/L)	12,334 (7007–17,763)	12,370 (6871–17,024)	12,220 (7070–20,183)	0.550
Number of oocytes retrieved	6.0 (4.0–11.0)	7.0 (4.0–12.0)	6.0 (3.0–10.0)	0.173
Pregnancy rate in the fresh IVF cycle	20/70 (28.6%)	6/35 (17.1%)	14/35 (40.0%)	0.036 *
Live birth rate in the fresh IVF cycle	9/67 (13.4%)	3/35 (8.6%)	6/32 (18.8%)	0.287

Values are expressed as median (25–75th percentile) for continuous variables and as number (percentage) for categorical variables. PPOS: progestin-primed ovarian stimulation. * Statistically significant.

**Table 5:** Characteristics and pregnancy outcomes between groups with serum CA-125 level in frozen embryo transfer cycles.

Parameters	Serum Level in the Mid-Cycle <35 IU/L (n = 84)	Serum Level in the Mid-Cycle ≥35 IU/L (n = 57)	Odds Ratio (95% Confidence Interval)	*p*-Value
Age of women at the time of transfer (years)	36.0 (34.0–37.0)	35.5 (33.0–37.0)	---	0.576
Body mass index (kg/m^2^)	20.5 (19.5–22.4)	21.2 (19.8–23.3)	---	0.274
Endometrial thickness (mm)	10.6 (9.2–12.3)	10.4 (9.0–12.3)	---	0.476
Type of cycles			---	0.413
Natural cycle	51 (60.1%)	33 (57.9%)		
Hormone replacement	26 (31.0%)	22 (38.6%)		
Letrozole-induced	4 (4.8%)	2 (3.5%)		
Pregnancy rate in frozen embryo transfer cycle	34/81 (42.0%)	20/56 (35.7%)	0.750 (0.295–1.907)	0.479
Live birth rate in frozen embryo transfer cycles	21/73 (28.8%)	10/52 (19.2%)	0.934 (0.429–2.036)	0.293

Values are expressed as median (25–75th percentile) for continuous variables and as number (percentage) for categorical variables.

**Table 6:** Effect of endometriosis surgery on serum CA-125 and AMH levels.

	Endometriosis Surgery (n = 152)	No Endometriosis Surgery (n = 82)	*p*-Value
Antral follicle count	7.0 (4.0–11.0)	8.0 (6.0–12.3)	0.650
Anti-Mullerian hormone level (ng/mL)	1.3 (0.7–2.4)	2.3 (1.1–4.0)	0.013 *
Baseline serum CA-125 level (IU/L) (cases with existing endometrioma excluded)	39.7 (19.2–79.4)	35.5 (23.0–58.5)	0.600
Serum CA-125 level on the trigger day (IU/L) (cases with existing endometrioma excluded)	29.5 (18.4–52.1)	32.6 (18.9–53.2)	0.761
Ovarian stimulation protocol			
GnRH antagonist	106/152 (69.7)	56/82 (68.3)	0.883
PPOS	46/152 (30.2)	26 (31.7)	
Total dose of gonadotrophins (IU)	3000 (2475–3600)	2700 (2250–3300)	0.040 *
Number of oocytes retrieved	6.0 (3.0–10.0)	6.5 (4.0–13.0)	0.094
Fresh embryo transfer	48/152	22/82	0.553
Pregnancy rate in fresh IVF cycle	16/48 (33.3)	4/22 (18.2)	0.170
Live birth rate in fresh IVF cycle	9/46 (19.6)	0/21 (0)	0.028 *

Values are expressed as median (25–75th percentile) for continuous variables and as number (percentage) for categorical variables. PPOS: progestin-primed ovarian stimulation. * Statistically significant.

## Data Availability

The datasets of this research are accessible from the corresponding author on requests.

## Supplementary Materials

The following supporting information can be downloaded at: https://www.mdpi.com/article/10.3390/medicina62010053/s1, Table S1. Characteristics and pregnancy outcome between groups with different stimulation protocols. Table S2. Pregnancy outcome between groups with serum CA-125 level on the trigger day.

## Abbreviations

The following abbreviations are used in this manuscript:

IVF	In vitro fertilization
GnRH	Gonadotrophin releasing hormone
LH	Luteinising hormone
PPOS	Progestin-primed ovarian stimulation
HCG	Human chorionic gonadotrophin
AMH	Anti-mullerian hormone
