# Supplementary material for: The Significance of Serum CA-125 Level on the Live Birth Rates of In Vitro Fertilisation in Women with Endometriosis
Source: Medicina (Kaunas). 2025 Dec 26;62(1):53. doi: 10.3390/medicina62010053 (PMC12842613; doi:10.3390/medicina62010053)
Supplement: Supplementary file 1 [file medicina-62-00053-s001.zip › medicina-3968322-supplementary.pdf]

medicina

MDPI

Supplementary Materials

Table S1. Characteristics and pregnancy outcome between groups with different stimulation protocols.

Parameters	GnRH Antagonist Protocol (n = 160)	PPOS Protocol (n = 74)	*p*-Value
Age of women (years)	36.0 (34.0–37.0)	35.5 (33.0–37.3)	0.567
Body mass index (kg/m^2)	21.1 (19.6–22.9)	21.2 (19.6–23.2)	0.831
Duration of infertility (years)	4.0 (2.0–5.0)	3.0 (2.0–4.3)	0.049 *
Antral follicle count	7.0 (4.0–11.0)	9.0 (6.8–12.0)	0.017 *
Anti-Mullerian hormone level (ng/mL)	1.1 (0.6–2.3)	1.9 (1.0–3.6)	0.003 *
Presence of endometrioma during ovarian stimulation	91/148 (61.5)	38/68 (55.9)	0.376
Size of endometrioma if any (mm)	17.0 (0–27.0)	11.0 (0–22.5)	0.133
CA-125 level at baseline (IU/L)	49.2 (29.2–103.0)	50.6 (23.6–107.6)	0.228
CA-125 level on the trigger day (IU/L)	39.8 (21.8–74.6)	29.1 (16.3–55.1)	0.004 *
Total dose of gonadotropins (IU)	3000 (2475–3600)	2700 (2156–3188)	0.001 *
Peak serum estradiol level (pmol/L)	11,219.5 (6625.8–17,104.8)	14,266.5 (7081.8–19,222.0)	0.128
Number of oocytes retrieved	6.0 (3.3–10.0)	7.0 (4.0–13.3)	0.118
Pregnancy rate in the frozen embryo transfer cycle	30/82 (36.6)	22/55 (40.0)	0.857
Live birth rate in the frozen embryo transfer cycle	20/76 (26.3)	11/49 (22.4)	1.000

Values are expressed as median (25–75th percentile) for continuous variables and as number (percentage) for categorical variables. PPOS: progestin-primed ovarian stimulation. * Statistically significant.

Table S2. Pregnancy outcome between groups with serum CA-125 level on the trigger day.

	<35 IU/L (n = 117)	>35 IU/L (n = 117)	Odds Ratio (95% Confidence Interval)	*p*-Value	Adjusted Odds ratio (95% Confidence Interval) #	*p*-Value
Pregnancy rate in the fresh IVF cycle	6/35 (17.1%)	14/35 (40.0%)	3.383 (1.111–10.299)	0.036 *	4.126 (1.241–13.720)	0.021 *
Live birth rate in the fresh IVF cycle	3/35 (8.6%)	6/32 (18.8%)	2.560 (0.582–11.261)	0.287	---	---

* Statistically significant. # Adjusted for antral follicle count.
